# Vegetation Types Mapping Using Multi-Temporal Landsat Images in the Google Earth Engine Platform

**DOI:** 10.3390/rs13224683

**Published:** 2021-11-19

**Authors:** Masoumeh Aghababaei, Ataollah Ebrahimi, Ali Asghar Naghipour, Esmaeil Asadi, Jochem Verrelst

**Affiliations:** 1 Department of Range and Watershed Management, Faculty of Natural Resources and Earth Sciences, Shahrekord University, Shahrekord 8818634141, Iran; 2 Image Processing Laboratory (IPL), Parc Científic, Universitat de Valencia, 46980 Paterna, Spain

**Keywords:** vegetation types classification, multi-temporal images, machine learning, Google Earth Engine, NDVI

## Abstract

Vegetation Types (VTs) are important managerial units, and their identification serves as essential tools for the conservation of land covers. Despite a long history of Earth observation applications to assess and monitor land covers, the quantitative detection of sparse VTs remains problematic, especially in arid and semiarid areas. This research aimed to identify appropriate multi-temporal datasets to improve the accuracy of VTs classification in a heterogeneous landscape in Central Zagros, Iran. To do so, first the Normalized Difference Vegetation Index (NDVI) temporal profile of each VT was identified in the study area for the period of 2018, 2019, and 2020. This data revealed strong seasonal phenological patterns and key periods of VTs separation. It led us to select the optimal time series images to be used in the VTs classification. We then compared single-date and multi-temporal datasets of Landsat 8 images within the Google Earth Engine (GEE) platform as the input to the Random Forest classifier for VTs detection. The single-date classification gave a median Overall Kappa (OK) and Overall Accuracy (OA) of 51% and 64%, respectively. Instead, using multi-temporal images led to an overall kappa accuracy of 74% and an overall accuracy of 81%. Thus, the exploitation of multi-temporal datasets favored accurate VTs classification. In addition, the presented results underline that available open access cloud-computing platforms such as the GEE facilitates identifying optimal periods and multitemporal imagery for VTs classification.

## 1 Introduction

Optical Earth observation (EO) data form the basis of land cover monitoring and mapping to obtain periodic, rapid, and accurate data [1]. Vegetation Types (VTs) mapping and analysis using EO data are essential for the management and conservation of natural resources and landscapes [2] as well as for the evaluation of ecosystem services [3,4]. VTs are defined as the distinctive kinds of land that differ from other kinds of land in the ability to produce distinctive types and amounts of vegetation [5]. Moreover, VTs describe the potential plant species that occur at a site with similar ecological responses to natural disturbances and management actions [6]. For instance, VTs descriptions inform managers about what kind of changes can be expected in response to management or disturbances and provide a reference for interpreting land cover data.

Despite the advantages of using EO data, processing satellite data to map VTs in heterogeneous landscapes poses multiple challenges [7]. Generally, VTs form complex yet related spatial structures within the heterogeneous landscape, and due to low inter-class separability lead to similar spectral responses. The production of reliable and accurate VTs maps in heterogeneous landscapes is typically based on the classification of raw satellite imagery. Spatial and temporal resolutions of spectral imagery are often inadequate to classify small-structured landscapes with diverse VTs, leading to a low classification accuracy [8]. Therefore, these heterogeneous plant covers impose challenges to spectral classification methods, especially when relying solely on single-date EO imagery data [9]. At the same time, multi-temporal images can play an important role in the VTs classification accuracy, as they provide data on distinct stages of the vegetation phenology [10]. This phenology information can thus be used for selecting the key periods (dates) of VTs separation and the optimal time series dataset in the VTs classification. Recent studies have highlighted the advantages of time series of EO data for mapping plant covers, not directly from specific plant species spectral reflectance, but indirectly from phenology seasonality [11,12]. Seasonal time series data embed the temporal aspects of natural phenomena on the land surface, which are highly desired by researchers and extremely helpful for discriminating different land cover types and vegetation classification [13]. Unique seasonal “signatures" of distinct VTs become critically important for discriminating between plant species and communities. An important consideration is the choice of a time window over which a seasonal curve is reliably representative of vegetation dynamics–that is typically the start and the end of the growing season. For instance, in the analysis of ecosystem properties in a northern US forest region, the seasonally averaged NDVI explained ~75% accuracy, while the single-date NDVI only explained 52% [14].

Multi-temporal Landsat image analyses have increased substantially since 2008. When it comes to vegetation mapping, all available Landsat images can be used to increase the number of good quality observations in a year [15]. Exploiting multi-temporal Landsat data would reduce the effects of poor-quality observations (affected by clouds, cloud shadows, and terrain shadows), and better capture phenological information of VTs in the classification [16]. The Google Earth Engine (GEE) hosts and stores satellite imagery in a public data archive that includes historical Earth images covering more than forty years. Regarding the processing of multi-temporal datasets, the GEE platform facilitates researchers to select and process large volumes of data [17]. Thanks to its open access, users can analyze all available remotely-sensed images using a web-based Integrated Development Environment (IDE) code editor without downloading these images to the local machine [18]. In addition, the cloud-based platform provides basic calculation functions for vector and raster data. Its high computational power offers land mapping approaches at national, intercontinental, and even global levels. GEE has been extensively employed in multiple data processing applications and environmental studies, such as forest degradation [15], cropland classification [19], urban land mapping [20], and green LAI mapping [21]. When relying on temporal data for land cover classification, the first critical step to take is the selection and combination of optimal time series datasets [22]. Some studies simply selected as many multi-temporal images, without concerns for the effects of different seasons or what vegetation spectral behavior might have on classification accuracy [23].

Overall, while using satellite imagery has been addressed relatively well in image classification, the process of vegetation cover classification is a more challenging and complex process. This is especially the case where VTs as a subclass of rangeland cover are concerned. Subclasses of vegetation cover are more spectrally similar than that of a higher hierarchical land cover types. To overcome this, images acquired at different dates during VTs growth periods are required to accurately identify and discriminate VTs. Our analysis provides insights into whether the use of an optimal multi-temporal dataset of Landsat OLI-8 images is sufficient to accurately classify VTs across heterogeneous rangelands at the landscape level. We chose a heterogeneous landscape in the southwest of Iran as our study area to cover different distinguishable VTs and their ecological significance. This research focuses on the VTs classification process using an optimal time series dataset derived from the NDVI temporal profiles and plant species' spectral behavior for the period of 2018, 2019, and 2020. In addition, we applied the machine learning classifier Random Forest to compare the VTs classification accuracy of the single-date and multi-temporal Landsat 8 images. This study will eventually provide insights into selecting the time series dataset for optimized VTs mapping in heterogeneous landscapes.

## 2 Materials and Methods

### 2.1 Study Area

**Figure 1:**
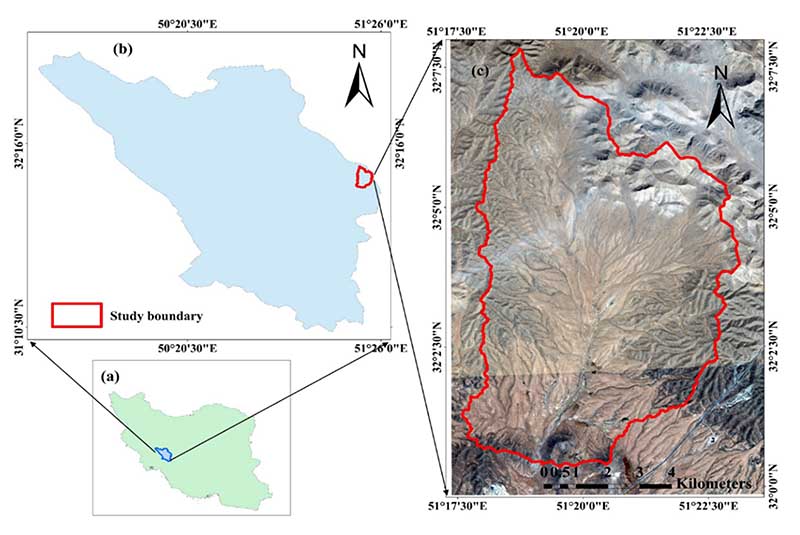
The location of the study area (**a**)—Iran border; (**b**)—Chaharmahal-Va Bakhtiari border; and (**c**)—Study area border (Marjan).

The semi-steppe Marjan rangelands is located within the Chaharmahal-Va Bakhtiari province in southwest Iran. The area covers 7736 ha, extending from 32º07’40” to 32º0’20”N and 51º17’30” to 51º23’00”E (Figure 1). This area with warm and dry summers and temperate and cold winters is considered an arid area with an average annual rainfall (1988-2020) of 200 mm. Despite its low annual rainfall (200 mm), due to appropriately implemented management practices, much of the study area has vegetation with a suitable canopy cover, whereby shrubs and perennial grasses dominate. VTs can be straightforwardly observed in this area due to relatively sharp borders and narrow ecotones between them.

### 2.2 Field Measurements of VTs

**Figure 2:**
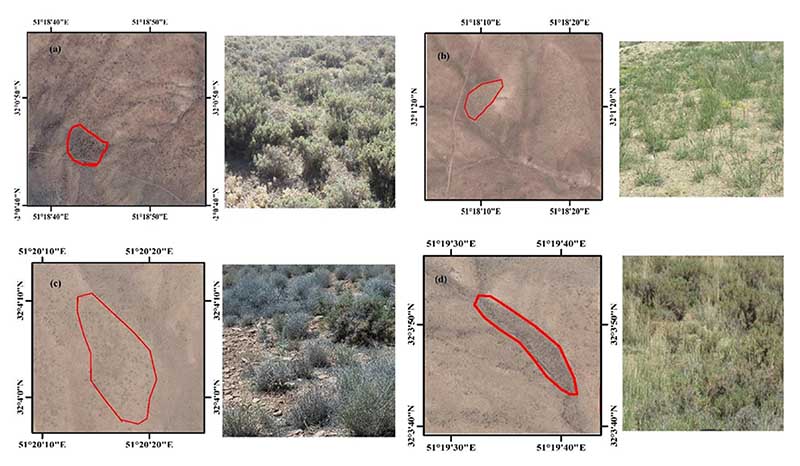
The location of VTs in Google Earth images and the corresponding field photos. (**a**)—VT1 (As ve); (**b**)—VT2 (Br to); (**c**)—VT3 (Sc or); and (**d**)—VT4 (As ve-Br to).

**Figure 3:**
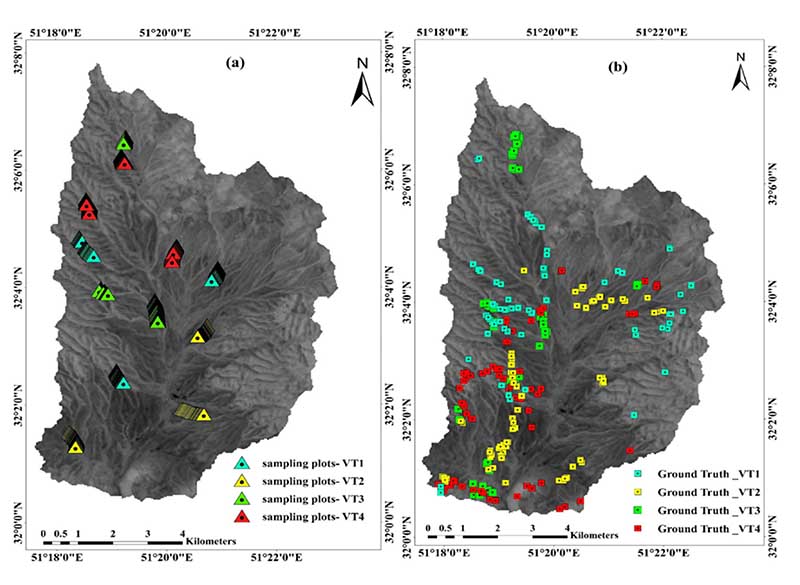
Study area boundary. (**a**)—Distribution of the field canopy cover sampling plots. (**b**)—The set of sampling points of VTs recorded in the field, which later divided into two groups of training and verification samples.

Four VT classes were identified in the study area (Figure 2), namely, (1) VT1 (*Astragalus verus* Olivier), (2) VT2 (*Bromus tomentellus* Boiss), (3) VT3 (*Scariola orientalis* Sojak), and (4) VT4 (*Astragalus verus* Olivier—*Bromus tomentellus* Boiss). The canopy cover data could potentially be used to identify VTs from structural, compositional, or a combination of both, the so-called physiognomic-floristic classification, to have a sound and accurate perspective on VTs. We sampled the four identified VTs using three replicates, in each of which the canopy cover was sampled along three transects of 100 m that were evenly distributed throughout the study area (Figure 3a). The sampling was systematic randomly (the first node was selected systematically, but the rest were randomly distributed along the transects). We collected a species-based canopy cover within each quadrat. In each VT, the canopy cover percentage was calculated, and the VTs were named according to their dominant floristic composition (Table 1). For this purpose, first the dominant plant species of each VT was identified, and then its accompanying species was determined with having 50% or more canopy cover of a previously dominant species cover. Thus, each VT was named based on a physiognomic-floristic method.

### 2.3 Spectral Time Series Landsat Data and NDVI Spectral Curve

The GEE platform was used to obtain the collection of Landsat 8 time series images (1 January 2018 to 31 December 2020) to accurately identify and classify VTs. Within GEE we selected Top of Atmosphere (TOA) reflectance (ee. Image Collection (‘LAND-SAT/LC08/C01/T1_SR')) and less than 5% cloud coverage (ee. Filter. Less than (‘CLOUD_COVER', 5)) Landsat 8 images. So, only images with less than 5% cloud cover are included. Thereby, some months of the year are excluded due to persistent cloudiness. A total of 36 cloudless dates were extracted for this study (Table 2).

We processed all the available collection of the Landsat 8 time-series images in the GEE to generate the NDVI spectral curve (Equation (1)). The NDVI is significantly related to the radiation absorbed by actively growing plants; vegetation absorbs strongly a red portion of the spectrum and reflects strongly in the near-infrared part of the spectrum:

(1) $$\text{NDVI}=\frac{\left(\text{NIR}-\text{Red}\right)}{\left(\text{NIR}+\text{Red}\right)}$$

where RED is the reflectance in the red band, and NIR is the reflectance in the near-infrared band [24].

In this research, by analyzing the NDVI temporal profile and plant species’ spectral behavior at different growth periods, we identified a dataset of an optimal combination of multi-temporal images (time-series dataset) as input for classification. In other words, we only used this index to identify optimal multi-time images for vegetation classification and did not use the NDVI as input for classification.

To determine the NDVI values for each VT, the NDVI values were extracted from the canopy cover sampling plots (Figure 3a). The corresponding values of the NDVI map were extracted as a table, and the NDVI diagrams of each VT for the period of 2018, 2019, and 2020 were drawn separately. Then, by analyzing the NDVI diagram of each year and the spectral behavior pattern of each VT in different growth periods, the best combination of multi-temporal images was selected for an accurate separation and classification of VTs.

### 2.4 Methodology

#### 2.4.1 Field Samples

After distinguishing the dominant VTs within the area, for each identified VT, 75 sample points were recorded by field excursion. The XY position of each representative VTs point was recorded using a Garmin eTrex 32× Handheld GPS (Figure 3b). In total, 300 sample points were recorded for the four VTs (Figure 1). The sample points were then randomly divided into two groups of 120 points (40%) used for classification as the “training samples” and 180 points (60%) used for the validation of the classification results as the “verification samples”.

#### 2.4.2 VTs Classification with Multi-Temporal Images

Multiple classification algorithms have been applied in land cover mapping studies, such as decision trees [25], artificial neural networks [26], random forest [23], and support vector machines [27]. Among these algorithms, the RF algorithm is considered one of the most powerful and robust machine learning methods [16,28,29]. The RF algorithm was therefore chosen as the preferred classifier. Accordingly, after selecting the optimal multi-temporal images with aggregation in the layers used (Collection), we used the RF algorithm to classify and map VTs. Bands 2–7 were also defined as the best band composition for classifying VTs. Bands uninformative for VTs mapping, such as thermal-TIR, coastal aerosol, and the cirrus bands, were excluded [30].

#### 2.4.3 Prediction Assessment and Statistical Comparison of Classifications

**Figure 4:**
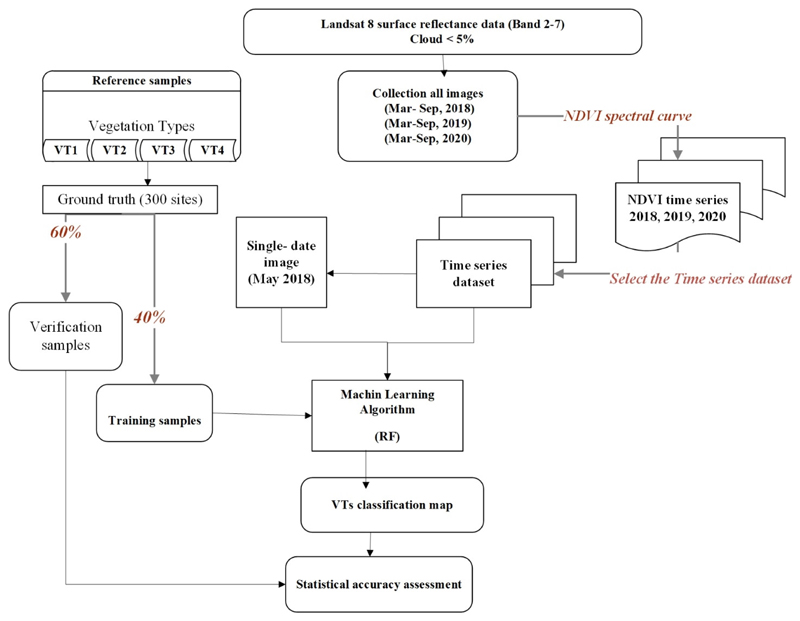
Workflow of VTs classification through selecting the optimal collection multi-temporal images with the RF classification algorithm.

For the classification process, the mapping accuracy was evaluated by means of the confusion matrix resulting from crossing the ground truth image of the “verification samples” and the outcome map of the classification process. Other accuracy indices to assess the performance of the classification include the Overall Accuracy (OA), Overall Kappa (OK), Kappa Index of Agreement (KIA), User’s Accuracy (UA), and Producer’s Accuracy (PA). As the confusion matrix only gives the performances of VTs maps based on validation samples, we additionally computed the Friedman test. This test enabled us to assess whether there was a statistically significant difference between single-date images and multi-temporal images in VTs classification. Figure 4 shows the conducted workflow to assess the optimal multi-temporal images for VTs classification. To focus on the effect of image selection on VTs classification, we selected all the Landsat 8 atmospherically corrected surface reflectance with less than 5% of cloud coverage scenes available on the GEE platform for the years 2018, 2019, and 2020 (encompassed the images from March to September). The NDVI values were extracted from sampling plots, and the NDVI temporal profiles of each VT at different growth periods (for 2018-2020) were drawn separately. A dataset of an optimal combination of multi-temporal images was selected, and with the purpose of investigating the effect of using multi-temporal images as opposed to using spectra from a single image, the May 2018 image served as a reference for the classification accuracy. For the RF classification, the collected 300 sample points were divided into two groups of 120 points (40%) used for classification as the “training samples” and 180 points (60%) used for the validation of the classification results as the “verification samples”. Finally, a statistical comparison was performed to assess the classification accuracy between single-date images and multi-temporal images in VTs classification.

## Results

### 3.1 NDVI Values Profile Results

**Figure 5:**
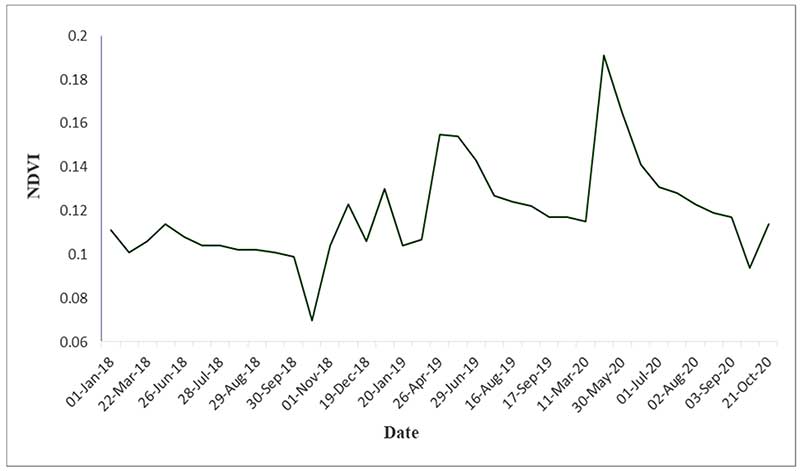
The NDVI time series profile of Landsat 8 images for the period 1 January 2018 to 21 October 2020.

Figure 5 shows the total NDVI index temporal profile for the years 2018, 2019, and 2020. In this profile, the pattern and trend to NDVI changes can be observed. The maximum NDVI values can be observed in spring, which coincides with the beginning of the vegetative growth of plant species. The minimum NDVI values are related to the dry seasons of the region, i.e., the summer and autumn seasons.

### 3.2 Select the Time-Series Dataset

**Figure 6:**
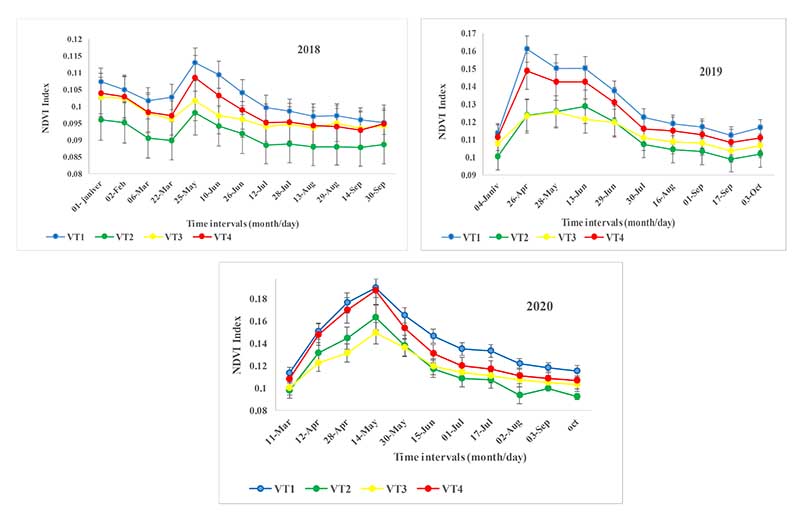
The NDVI temporal profile and error bars for each VT class for the years 2018-2020.

Using the NDVI temporal profile for specified time intervals, the plant species spectral behavior can be analyzed at different growth periods, and an optimal combination of multi-temporal images (time series dataset) can be selected to improve the VTs classification. The NDVI temporal profile and error bars for each VT class are shown in Figure 6; all four VTs reveal a similar spectral shape, but their values are different. According to Figure 6, VT1 and VT4 have the highest spectral reflectance and NDVI values in all three years. However, VT 2 and VT3 express distinct spectral behavior and NDVI values in each year depending on the environmental and climatic conditions. During spring, which coincides with the beginning growth and maximum plant species growth, there is less overlap of NDVI values between plant species. This season and growth period mark the highest degree of separation between VTs. However, in the dry seasons, i.e., summer and autumn, which are the deciduous stage of most plant species, the NDVI temporal profiles show an identical pattern and the most similar spectral response for VTs, leading to a low separation between VTs.

In general, the highest NDVI value change occurs every three years between April and June. This multi-temporal time window is then used to optimize the classification of different VTs.

### 3.3 VTs Classification

**Figure 7:**
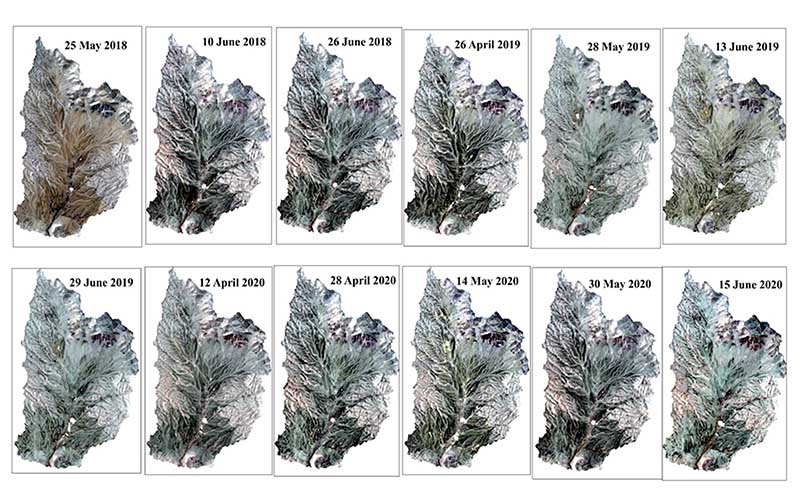
A collection of RGB images from the optimal multi-temporal images for VT classification.

As shown in Figure 7, after analyzing the NDVI temporal profiles and plant species’ spectral behavior at different growth periods, the multi-temporal images with the most distinct spectral response (optimal time series dataset) were selected for VTs classification.

**Figure 8:**
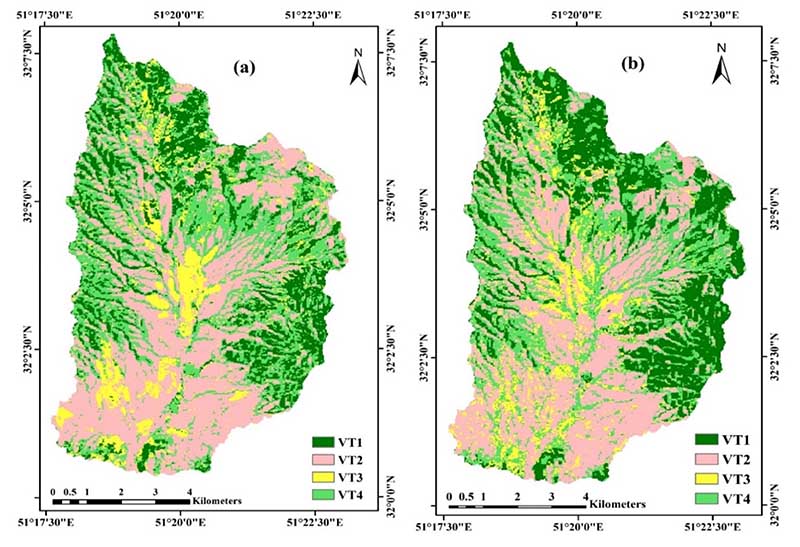
VTs classification maps using the RF algorithm (**a**)—VTs classification map obtained from single-date images. (**b)—**VTs classification map obtained from multi-temporal images.

After selecting the dataset of an optimal combination of multi-temporal images and creating an image collection (Band 2-7 for each image, in other words, 72 bands) using the RF algorithm, VTs classification was performed (Figure 8b). The single image of May 2018 chosen as the reference for classification comparison is also shown in Figure 8a.

### 3.4 Comparing Single-Date Image and Multi-Temporal Images in VTs Classification

Table 3 gives the results of the confusion matrices for the VTs classifications achieved from single-date images and multi-temporal images classification. In this table, the OA and OK of each classification process are reported. In addition, the PA, UA, and KIA for each VT are reported. When a single image was applied, VT1 had the highest PA and UA with 90% and 74%, respectively. However, VT2 led to the lowest PA with 34%. The overall kappa was 51%, and the overall accuracy was 64%.

Using the multi-temporal images led to the improvement of VTs classification accuracies. The performance of the multi-temporal images showed an overall kappa accuracy of 74% and an overall accuracy of 81%. The side-by-side comparison of the performance of single-date images and multi-temporal images revealed that multi-temporal images improved the OA by 17% and OK accuracy by 23% (Table 3).

### 3.5 Statistical Comparison

The statistical comparisons of multi-temporal images and single-data images for VTs classification using the Friedman test are shown in Table 4. After calculation of the PA, UA, and KIA, we used the Friedman test to examine whether the classification accuracy between single-data images and multi-temporal images is a statistically significant (sig < 0.05) difference. As shown in Table 4, the PA, UA, and KIA showed statistically significant differences on the VTs classification accuracy (*p* < 0.05).

## 4 Discussion

The construction of a fast, accurate, and simple model for extracting land cover information and VTs maps is of concern to natural resources managers and ecologists [31]. This study examined whether the optimal multi-temporal dataset of Landsat OLI-8 images is sufficient to accurately classify VTs across heterogeneous rangelands at the landscape level. After identification of distinct VTs in the study area, the canopy cover percentage was calculated and named according to its dominant floristic composition. Finally, four VTs classes were identified: VT1 is a shrubby species (*As ve*), VT2 is a tallgrass species (Br to), VT3 is semi-shrub species (Sc or), and VT4 is the combination of shrub and tallgrass species (*As ve-Br to*).

Field methods are a useful tool for accurate identification and classification of VTs, but these methods face limitations, and due to personnel, logistical, and budgetary limitations, field measurement methods cannot make repeated and simultaneous in situ observations of the heterogeneous landscapes [32]. The increasing availability of satellite data has provided free imagery with high spatial and spectral resolutions, such as Landsat 8, that are considered essential tools for land cover mapping [33]. However, the classification of VTs relying on a single-date Landsat image is challenging, especially in our heterogeneous

study area. This issue is particularly relevant to VTs, thus phenological data become important in the land cover mapping of the VTs distribution and subsequently in their classification, while single-date image assessments may not accurately represent annual changes and discriminate vegetation [23].

### 4.1 NDVI Temporal Profiles

According to the NDVI temporal profile in Figure 5, maximum NDVI values can be observed in spring. In addition, the role of the VTs phenology should be discussed. As shown in Figure 6, the most informative temporal window among the VTs classes was observed for the period of April through June. The most critical months for VTs discrimination were when minimal reflectance values were observed (winter and summer seasons) and when the NDVI reflectance was similar among the VTs. Given that the predominant VTs in the study area are shrubs (*As vr*), semi-shrubs (*Sc or*), and grasses (*Br to*), shrub species, due to their higher canopy cover percentage, have a higher NDVI value than the grasses and semi-shrubs species in the three years of 2018, 2019, and 2020.

In addition, due to the low precipitation in the area in 2018 (170 mm), VT2 with dominant grass species (*Br to*) is not drought resistant and shows the lowest vegetative growth rate, leading to the lowest NDVI value. Other VTs (*As ve* and *Sc or*) are more resistant to drought due to shrubby and semi-shrub species dominance or compositional variation, and have maintained their canopy cover, thus maintaining a higher NDVI value than the VT2. The amount of precipitation somewhat increased in 2019 and 2020 (220 and 210 mm, respectively), which meant that the VT2 dominant grass species had better vegetative growth than semi-shrubs and had a higher NDVI value in early spring. However, the high palatability of these grass species, as opposed to shrubby and semi-shrub species, favors intensive grazing, and the canopy cover starts to decrease starting from late spring onwards. Likewise, the grazing provoked a decrease in NDVI values (Figure 6). Therefore, VTs’ spectral behavior is different in the growth period, and this is the most important factor for selecting the time window for identifying and separating shrubs and grasses.

### 4.2 Mapping VTs

Landsat OLI-8 images were used over a period of three years from 2018 to 2020. The first step was to select the optimal multi-temporal images for VTs classification. By analyzing the NDVI temporal profile and plant species’ spectral behavior, we identified the optimal combination of multi-temporal images as input for VTs classification.

The second critical step was to determine how to use these multi-temporal datasets for VTs classification. Obviously, such large data volumes are not easy to handle and analyze. The GEE platform allows to synchronize all the Landsat 8 data and then establish a high-quality, multi-temporal dataset using codes already provided [34]. Such an approach not only provides cloud-free, multi-temporal images, but also makes it easier to analyze vast amounts of multi-temporal images, thus reducing the need to produce individual maps for all of the available images [21].

For instance, by aiming to identify the potential impact of different sampling times on the estimation of rangeland monitoring, [35] reported that the GEE platform is an ideal testbed and critical component of a system that can be used to provide land cover information. In addition, [36] reported that on the GEE platform, hundreds of images can be rapidly processed. Using the median composition method, the input images are created in a pixelwise manner by taking the median value from all pixels of the image collection. The advantage of this method is the significant reduction of data volume, resulting in a faster and easier analysis.

The RF algorithm was chosen for VTs classes mapping. The classification algorithm’s success for land cover classification depends on many factors, such as the characteristics of the study area, the classification system, satellite images, and the use of a multi-temporal dataset [27]. The RF algorithm is a tree-based machine learning method that leverages the power of multiple decision trees for making decisions and is suitable for situations when we have a large dataset [37]. In a related study, the impact of multi-temporal images (across months and years) for rangeland monitoring was analyzed in the GEE platform [35]. The authors observed that the RF algorithm yielded the most accurate results, and the other two algorithms (Perceptron and Continuous Naive Bayes) produced considerably more errors in the overall model performance.

### 4.3 The Roles of Multi-Temporal Satellite Imagery in VTs Classification

We analyzed two models for optimal VTs classification in this study. The first model includes a single-date image (May 2018) from Landsat OLI-8 images with an RF classifier. The overall classification accuracy (64%) and overall kappa (51%) were obtained in the first model (Table 3).

The second model is based on the optimal multi-temporal images (2018, 2019, and 2020) from Landsat OLI-8 images with an RF classifier. While development of a multi-temporal dataset is often time consuming and requires optimization of the plant species’ phenological behavior, it is the most important step to identifying an optimal multi-temporal dataset to represent the different VTs between different kinds of land cover. This research introduces an optimal multi-temporal dataset, which is valuable in improving VTs classification accuracy. The results of the second model showed that combinations of distinct multi-temporal datasets can improve the OA (17%) and OK (23%).

The usage of multi-temporal satellite imagery provides important information for VTs mapping and classification. In the multi-temporal satellite images, using plant species’ phenological behavior during the growing season can be selected as the best feature space in the temporal domain, so that the separation degree increases as much as possible between different VTs. In a related study about using multi-temporal images in classification, Stumpf [12] found that for the spatial monitoring of grassland management, the spectral time series from satellite imagery allows progressing phenological stages to be detected and can be used for the multi-temporal dataset for grasslands classification and management.

The produced maps were validated against ground truth data, the so-called verification samples, by computing the OA (Figure 8b). The resulting maps from multi-temporal Landsat 8 images produced the highest OK (74%) and OA (81%). It is yet to be questioned whether this accuracy is high enough for their use in practical applications. According to the Land Use/Land Cover classification system with remotely sensed data developed by Anderson in 1976 (American Geological Survey), nine main classes were identified, including Urban or Built-up Land, Agricultural Land, Rangeland, Forest Land, Water, Wetland, Barren Land, Tundra, and Perennial Snow or Ice. In addition, subclasses have been introduced for each of these major classes. So far, most of the land classification process have been based on the main classes, such as Feng [38], Pflugmacher [30], and Macintyre [14]. However, our study differs in that the main purpose is to optimize the classification process for rangeland VTs subclasses. When it comes to the mapping of rangeland VTs, they are characterized by a similar spectral behavior (low interclass separability) and a complex spatial structure. The separation of VTs is therefore a hard task, and our obtained OA of 81% can be considered as sufficiently satisfactory.

## 5 Conclusions

The identification and classification of VTs in a spectrally heterogeneous landscape is among the most challenging tasks in satellite image classification. In this study, we conducted a detailed experiment on how to improve image classification accuracy by integrating multi-temporal images. The presented results suggested that single-date images do not lead to a proper identification of VTs. Instead, our results underpin that the development of an accurate VTs map is feasible in a heterogeneous landscape when a dataset of an optimal combination of multi-temporal images is entered into an RF machine learning classifier. To do so, stacking and filtering the multi-temporal images based on the cloud cover threshold are required. By analyzing the NDVI temporal profile and plant species’ spectral behavior at different growth periods, we identified the multi-temporal images with the most distinct spectral response as input for RF classification. The classification results revealed that multi-temporal satellite imagery provides important information for VTs detection and mapping. Compared to single-date images, it led to an OA and OK improvement of 17% and 23%, respectively.

When it comes to perspectives for future work, cloud-computing platforms such as the GEE opened opportunities to quickly identify optimal periods and time series dates for VTs classification. While the multi-temporal images dataset is most promising for VTs classification, further research should focus on the exploration of the relationships between novel EO data processing techniques and dynamic VTs mapping.

## Figures and Tables

**Table 1:** The identified VTs and their vegetational characteristics in the study area.

**Code**	Dominant Species *	**Dominant Life Form**	Accompanied Species *	**Dominant Soil Type**
VT1	*Astragalus verus* Olivier. (*As ve*). (23.4%)	Shrub	*Scariola orientalis* (Boiss) Sojak. (2.5%) *Alyssum linifolium Steph.* ex Wild. (2%) *Heteranthelium piliferum Hochst.* ex Jaub. (1.8%) *Astragalus macropelmatus* Bunge. (1.3%) *Acanthophyllum spinosum* (Desf.) C.A.Mey. (0.8%)	Sandy loamy to loamy clay
VT2	*Bromus tomentellus* Boiss. (*Br to).* (8.9%)	Tallgrass	*Phlomis olivieri* Benth. (2.5%) *Stipa hohenackeriana* Trin & Rupr. (2%) *Achillea wilhelmsii* C. Koch, L. (1.8%) *Centaurea aucheri* (DC.) Wagenitz. (1.2%) *Gypsophila struthium.* (1%)	Loamy and silty loamy
VT3	*Scariola orientalis* (Boiss.) Sojak. (Sc or). (9.25%)	Semi-shrub	*Noaea mucronata* (Forsk.) Aschers et. Sch. (2.5%) *Polygonum aridum* Boiss. & Hausskn. (1.5%) *Stachys inflata* Benth. (1.2%) *Tragopogon longirostris* Bischoff ex Sch.Bip. (1%) *Chardinia orientalis* (L.) Kuntze. (0.5%)	Clay loam
VT4	*Astragalus verus* Olivier (8.6%)—*Bromus tomentellus* Boiss (5.4) (*As ve–Br to*)	Shrub-Tallgrass	*Euphorbia azerbajdzhanica* Bordz. (2%) *Phlomis persica* Boiss. (1.5%) *Turgenia latifolia* (L.) Hoffm. (1.5%) *Astragalus effusus* Bunge. (1.3%) *Cichorium intybus* L. (0.5%)	Loamy and silty loamy

* Canopy cover percentage of dominant and accompanied species that was calculated on transects.

**Table 2:** Landsat 8 images dates extracted in the GEE system.

**Year**	**Month/Day**	**Year**	**Month/Day**	**Year**	**Month/Day**
2018	1 January 2 February 6, 22 March 25 May 10, 26 June 12, 28 July 13, 29 August 14, 30 September 17 November 19 December	2019	20 January 26 April 28 May 13, 29 June 30 July 16 August 1, 17 September 19 October	2020	11 March 12, 28 April 14, 30 May 15 June 1, 17 July 18 August 3 September 21 October

**Table 3:** Confusion matrix results. Summary of the classification accuracy for each VT by single-date images and multi-temporal images.

**Confusion Matrix Results Based on Single-Date Image Classification**							
**Type**	**VT 1**	**VT 2**	**VT 3**	**VT 4**	**PA**	**UA**	**KIA**
VT1	10	0	0	4	90	74	65
VT 2	0	8	4	3	67	54	37
VT 3	0	3	7	1	59	64	51
VT 4	1	1	1	4	34	67	55
	Overall Kappa: 51%				Overall Accuracy: 64%		
**Confusion Matrix Results Based on Multi-Temporal Images Classification**							
**Type**	**VT 1**	**VT 2**	**VT 3**	**VT 4**	**PA**	**UA**	**KIA**
VT1	10	0	0	1	91	91	88
VT 2	0	10	3	1	84	72	61
VT 3	0	2	9	1	75	75	66
VT 4	1	0	0	9	75	90	86
	Overall Kappa: 74%				Overall Accuracy: 81%		

PA: Producer’s Accuracy %, UA: User’s Accuracy %, and KIA: Kappa Index of Agreement %.

**Table 4:** Results of the statistically significant comparison of multi-temporal images and single-date images in VTs classification.

VTs Accuracy	Sig
Producer’s Accuracy (PA)	0.038 *
User’s Accuracy (UA)	0.023 *
Kappa Index of Agreement (KIA)	0.038 *

The symbol “*” indicates that the difference is statistically significant because the significant level is 0.05.
